# Medical Journals

**Published:** 1860-07

**Authors:** 


MEDICAL JOURNALS.—We have received the first two numbers of the *Gazeta Medica do Porto*, a monthly periodical of medicine, surgery, pharmacy, and the accessory sciences, edited by Dr. José Fructuoso Ayres de Gouvêa Osorio. It is intended to represent the existing state of medical science in Portugal, and Dr. de Gouvêa Osorio has associated with him an able corps of collaborators in the persons of the different professors of the Medico-Chirurgical College of Porto.

Another monthly journal, we believe the fifth, has recently made its appearance in Georgia, under the title of the *Georgia Medical and Surgical Encyclopedia*, the May and June numbers of which are on our table. It is edited by Dr. H. N. Hollifield and Dr. Tom W. Newsome.

The *American Medical Times* is the title of a new weekly journal, the successor of the "New York Journal of Medicine," the first number of which was issued on the second of June of this year. It resembles in appearance very much the *Medical Times and Gazette* of London, and each number will consist of twenty-four quarto pages. Dr. Stephen Smith, so long and so honorably associated with the medical press, will retain the position of editor, and be assisted by Drs. Elisha Harris and G. F. Shrady. We wish the journal much success, and gladly place it upon our exchange list.

With the March issue, the *Peninsular and Independent Medical Journal* of Michigan ceased its existence, on account of the want of support from the profession of that State. Its editors, Drs. Palmer and Gunn, have long been connected with medical journalism, and we regret to find that we have lost them from our ranks.

Dr. McCormick has retired from the editorial chair of the *Pacific Medical and Surgical Journal;* and Dr. J. S. Wilson has become corresponding editor of the *Savannah Journal of Medicine.*

